
JOURNAL INFORMATION
==============================
NLM Title Abbreviation: Access Microbiol
Journal ID: Access Microbiol
NLM Journal ID: 101746927
Journal ID: acmi

Access Microbiology

EISSN: 2516-8290
Publisher: Microbiology Society

ARTICLE INFORMATION
==============================
PMCID: PMC7544068
PMID: 33062930
Article version: 1
Subjects: Abstracts from the Microbes in Medicine Meeting 2019

Oral Abstracts

Publication date: 2020
Electronic publication date: 2020 Jan 31
Volume: 2
Issue: 1
Electronic Location ID: po0001-po0006
Copyright: © 2020 The Authors
Copyright year: 2020
License: This is an open-access article distributed under the terms of the Creative Commons Attribution License.
License URL: https://creativecommons.org/licenses/by/4.0/

==============================

DOI: 10.1099/acmi.mim2019.po0001

Experimental evolution selects clinically relevant antibiotic resistance in biofilms but with collateral tradeoffs

http://jmmcr.microbiologyresearch.org

Trampari Eleftheria *
Holden Emma
Wickham Gregory
Ravi Anuradha
Prischi Filippo
Martins Leonardo de Oliveira
Savva George
Bavro Vassiliy
Webber Mark
Quadram Institute , Norwich , United Kingdom
University of Essex , Colchester , United Kingdom
*Correspondence: Eleftheria Trampari, eleftheria.trampari@quadram.ac.uk

DOI: 10.1099/acmi.mim2019.po0002

Factors governing orthologous RpoD and H-NS evolution in Salmonella enterica Serovar Typhimurium and Escherichia coli

http://jmmcr.microbiologyresearch.org

Mogre Aalap *
Dorman Charles
Trinity College Dublin , Dublin , Ireland
*Correspondence: Aalap Mogre, aalap.mogre@gmail.com

DOI: 10.1099/acmi.mim2019.po0003

Short-term consumption of a high-fat diet increases host susceptibility to Listeria monocytogenes infection

http://jmmcr.microbiologyresearch.org

Heras Vanessa *
Clooney Adam G.
Ryan Feargal J.
Cabrera-Rubio Raul
Casey Pat G.
Hueston Cara M.
Pinheiro Jorge
Rudkin Justine K.
Melgar Silvia
Cotter Paul
Hill Colin
Gahan Cormac
University College Cork , Cork , Ireland
APC Microbiome Ireland , Cork , Ireland
Moorepark Food Research Centre , Teagasc , Cork
*Correspondence: Vanessa Las Heras, vanessa.lasheras@ucc.ie

DOI: 10.1099/acmi.mim2019.po0004

Transposable temperate phages promote the evolution of divergent social strategies in Pseudomonas aeruginosa populations

http://jmmcr.microbiologyresearch.org

O'Brien Siobhan *
Kummerli Rolf
Paterson Steve
Winstanley Craig
Brockhurst Mike
University of Liverpool , Liverpool , United Kingdom
University of Zurich , Zurich , Switzerland
University of Sheffield , Sheffield , United Kingdom
*Correspondence: Siobhan O'Brien, siobhan.o-brien@liverpool.ac.uk

DOI: 10.1099/acmi.mim2019.po0005

Pharmacological modulation of the autophagy pathway enhances Mtb killing

http://jmmcr.microbiologyresearch.org

Quaid Kate Mc *
Keane Joseph
Kornfeld Hardy
O'Sullivan Mary
O'leary Seonadh
Trinity College Dublin , Dublin , Ireland
University of Massachusetts Medical School , Boston , USA
*Correspondence: Kate Mc Quaid, mcquaidk@tcd.ie

DOI: 10.1099/acmi.mim2019.po0006

The role of mature mycolic acids in mycobacterial biofilm formation and interactions with the human complement system

http://jmmcr.microbiologyresearch.org

Halkerston Rachel *
Davies Katherine
Tolley Howard
Keating Thomas
Taylor Stephen
Alderwick Luke
Bacon Joanna
Public Health England , Porton Down , Salisbury
University of Birmingham , Birmingham , United Kingdom
*Correspondence: Rachel Halkerston, rachel.halkerston@phe.gov.uk

